# Characterization of Effects of mTOR Inhibitors on Aging in *Caenorhabditis elegans*

**DOI:** 10.1093/gerona/glae196

**Published:** 2024-08-16

**Authors:** Aihan Zhang, Gadea Meecham-Garcia, Chiminh Nguyen Hong, Peiyun Xie, Carina C Kern, Bruce Zhang, Hannah Chapman, David Gems

**Affiliations:** Institute of Healthy Ageing, and Research Department of Genetics, Evolution and Environment, University College London, London, UK; Institute of Healthy Ageing, and Research Department of Genetics, Evolution and Environment, University College London, London, UK; Institute of Healthy Ageing, and Research Department of Genetics, Evolution and Environment, University College London, London, UK; Institute of Healthy Ageing, and Research Department of Genetics, Evolution and Environment, University College London, London, UK; Institute of Healthy Ageing, and Research Department of Genetics, Evolution and Environment, University College London, London, UK; Institute of Healthy Ageing, and Research Department of Genetics, Evolution and Environment, University College London, London, UK; Institute of Healthy Ageing, and Research Department of Genetics, Evolution and Environment, University College London, London, UK; Institute of Healthy Ageing, and Research Department of Genetics, Evolution and Environment, University College London, London, UK

**Keywords:** Age-related pathology, Lifespan, mTOR, Rapalog, Rapamycin

## Abstract

Pharmacological inhibition of the mechanistic target of rapamycin (mTOR) signaling pathway with rapamycin can extend lifespan in several organisms. Although this includes the nematode *Caenorhabditis elegans*, effects in this species are relatively weak and sometimes difficult to reproduce. Here we test effects of drug dosage and timing of delivery to establish the upper limits of its capacity to extend life, and investigate drug effects on age-related pathology and causes of mortality. Liposome-mediated rapamycin treatment throughout adulthood showed a dose-dependent effect, causing a maximal 21.9% increase in mean lifespan, but shortening of lifespan at the highest dose, suggesting drug toxicity. Rapamycin treatment of larvae delayed development, weakly reduced fertility and modestly extended lifespan. By contrast, treatment initiated later in life robustly increased lifespan, even from Day 16 (or ~70 years in human terms). The rapalog temsirolimus extended lifespan similarly to rapamycin, but effects of everolimus were weaker. As in mouse, rapamycin had mixed effects on age-related pathologies, inhibiting one (uterine tumor growth) but not several others, suggesting a segmental antigeroid effect. These findings should usefully inform future experimental studies with rapamycin and rapalogs in *C. elegans*.

The mechanistic target of rapamycin (mTOR) pathway is a central regulator of cellular growth and metabolism that also affects aging and age-related disease (1). Rapamycin, a macrolide inhibitor of mTOR, can extend lifespan in various organisms, including *Caenorhabditis elegans* (2), the fruit fly *Drosophila melanogaster* (3) and mice (4). In *C. elegans*, inhibiting insulin/IGF-1 signaling (IIS) can more than double *C. elegans* lifespan (5,6). By contrast, effects on lifespan of inhibiting mTOR signaling are typically more modest, for example, with increases in mean lifespan from *let-363* (mTOR) or *rsks-1* (S6 kinase, mediates effects of TORC1 on protein translation) RNAi ranging from +12%–47% (7), or for mutation of *rsks-1* from +12%–20% (8,9) (mutation of *let-363* causes mid-larval arrest) (10). In line with this, effects of rapamycin on *C. elegans* lifespan are usually relatively weak, with reported increases in mean lifespan ranging from +8%–50% (11–14), though in one case +116% (2). Moreover, the life-extending effect of rapamycin in *C. elegans* is sometimes difficult to reproduce (15); for example, recent work with the WormBot automated drug screening device (16) recorded no effect on lifespan (M.B. Lee, personal communication).

One aim of the present study is to maximize *C. elegans* lifespan extension by pharmacological inhibition of mTOR in order to facilitate future investigations. Here a limitation with rapamycin is its hydrophobicity which constrains drug delivery, for example, due to low solubility in the hydrophilic environment of NGM plates. Drug delivery to *C. elegans*, including that of rapamycin, can be aided by liposome encapsulation (15,17). This approach also requires far smaller amounts of drug than the standard method of delivery in agar plates, thereby reducing costs (which can otherwise be prohibitive for some compounds). Another possible approach to improve mTOR inhibition, pursued here, is to use rapalogs, rapamycin derivatives that are more soluble, such as temsirolimus and everolimus (18).

Another aim of this study is to deepen our understanding of how rapamycin affects aging in *C. elegans*, addressing two questions in particular. First, when during *C. elegans* life history are effects of rapamycin on lifespan exerted? In *Drosophila* and mouse, rapamycin treatment during development is sufficient to extend adult lifespan (19–21), and in mouse treatment from mid-life onwards or briefly in later life can also extend lifespan (4,22,23). Second, how does rapamycin affect diseases of aging and causes of death in *C. elegans*? In mice, rapamycin retards the development of many diseases of aging, but some it makes worse, for example, cataracts and testicular degeneration (24,25).

## Method

### Culture Methods and Strains

*Caenorhabditis elegans* maintenance was performed using standard protocols (26). Except where noted, all strains were grown at 20˚C on nematode growth media (NGM) plates seeded with *Escherichia coli* OP50 as a food source. In certain experiments (specified), *E. coli* OP50 bacterial lawns were exposed to an antibiotic (carbenicillin) to inhibit bacterial infection of *C. elegans* (15). An N2 hermaphrodite stock recently obtained from the Caenorhabditis Genetics Center was used as wild type (N2H) (27).

### Liposome-Mediated Compound Delivery

Liposomes were prepared using an Avanti Mini Extruder and L-α-phosphatidylcholine (L-α-PC) (Avanti Polar Lipids) (15,17). Liposomes were generated by passage through an extruder membrane with 100 nm diameter pores. To concentrate liposomes or separate them from free compound, liposome extruder purification (LEP) was used (28), which reduces the volume of a liposome suspension by using an extruder membrane with smaller (50 nm diameter) pores. Liposomes containing drug or fluorescent dye were delivered on the surface of small *E. coli* lawns (15). Concentrations of compounds used are listed in Table 1.

**Table 1.** Drugs Concentrations Used in Experiments Unless Otherwise Stated

Compound (Source)	Stock solution concentration, solvent	Final concentration
Carbenicillin (Fisher)	500 mM, MilliQ water	4 mM (within 10 mL NGM plate)
Rapamycin (Bio-Techne)	54.7 mM, DMSO	100 µM (within liposomes)
Everolimus (Stratech Scientific)	10 mM, DMSO	100 µM (within liposomes)
Temsirolimus (Stratech Scientific)	10 mM, DMSO	100 µM (within liposomes)
Texas Red (Invitrogen)	15 mM, MilliQ water	7.5 mM (within liposomes)
Uranine (Sigma-Aldrich)	300 mM, MilliQ water	150 mM (within liposomes)

*Notes*: DMSO = dimethylsulfoxide NGM = nematode growth medium. Fisher, Hampton, NHBio-Techne, Minneapolis, MNStratech Scientific, Ely, UKInvitrogen, Waltham, MASigma-Aldrich, St. Louis, MO.

### Microscopy

For epifluorescence imaging a Zeiss Axio Imager.Z2 microscope was used with a Hamamatsu digital camera C13440 ORCA-Flash4.0 V3 and Zen software. Live worms were viewed on 2% agar pads, anaesthetized with 10 μl 2 mM levamisole. For trials using uranine (fluorescein), green fluorescence was observed using a GFP filter (λ_ex_/λ_em_ 450–490 nm/500–550 nm; Filter Set 90 HE). For Texas red, red fluorescence was observed using a DsRed filter, (λ_ex_/λ_em_ 530–560 nm/590–650 nm; Filter Set 91 HE). Worm fluorescence was estimated as the mean pixel density of the worm image area minus that of the image background. Nematode volume was estimated by measuring length and mid-body width, and approximating animal morphology as a cylinder (*V* = *πr*^2^*h*).

### Survival Analysis

Nematodes were maintained at a density of 25–30 per plate, and transferred daily during the egg laying period, followed by every 6–7 days thereafter. The L4 stage was defined as Day 0. Mortality was scored every 1–2 days, with worms scored as alive if they showed any movement, either spontaneously or in response to gentle touch with a worm pick. Animals lost due to causes other than death from senescence (eg, death from internal hatching of larvae, or to desiccation on the Petri dish wall) were censored, and censor data used in statistical analysis (right censoring). Necropsy analysis to distinguish P and p corpses (29) was performed in specified trials. Raw mortality data for all trials is available in Ziehm tables, which apply minimal reporting standards (29) (Supplementary Data 1).

### Measurement of Nematode Age-Related Pathologies

High magnification Nomarski microscopy was used to image age-related pathologies on Days 1, 7, and 14 of adulthood (30,31). The pathologies examined reach their maximal extent by around Day 14 (30). Five pathological changes were measured: pharyngeal degeneration, gonadal atrophy and fragmentation, intestinal atrophy, uterine tumor development, and pseudocoelomic yolk pool accumulation.

Quantitative analysis was performed on intestinal atrophy and yolk pool accumulation. For the former, measurements were made of the diameter of the posterior intestine between the posterior gonad arm bend and the anus. From this the diameter of the intestinal lumen was subtracted, and the resulting value normalized to the overall body width, yielding an estimate of the cross-sectional width of the intestine relative to body size. Yolk pool accumulation was estimated by calculating the ratio of the yolk pool area to the body area within the field of view. Semi-quantitative scoring methods were used to assess the other three pathologies. Here image evaluation entailed assigning scores on a scale ranging from 1 to 5, with 1 representing a state indicative of youth and robust health, 2 denoting subtle signs of degeneration, 3 indicating distinct yet mild pathological manifestations, 4 signifying prominent pathology, and 5 representing very severe and maximal level of pathology (30,31).

### Statistical Analysis

All statistical tests were performed on raw data using GraphPad Prism 9.0 (GraphPad Software, Boston, MA) and JMP Pro 15 (JMP Statistical Discovery LLC, Cary, NC) unless otherwise stated, with the specific tests and post hoc corrections performed described in the figure legends. No statistical methods were used to predetermine sample size. The experiments were not randomized. The investigators were not blinded to allocation during experiments and outcome assessment.

## Results

### Identification of Rapamycin Dose With Maximal Effect on Lifespan

We first set out to establish the maximum achievable increase in *C. elegans* lifespan using rapamycin, delivered throughout adulthood, by administering the drug at a range of doses. Rapamycin was administered in liposomes (100 μM), delivered on the surface of the *E. coli* lawn, and given repeatedly during adulthood from L4 onwards, every 2 days until Day 8 of adulthood, and every 4 days thereafter (15). Given that rapamycin has limited solubility, drug dose was ramped up by increasing the quantity of liposomes administered, as follows: 50 μl = 1× dose, 100 μl = 2×, 200 μl = 4×, and 100 μl of 5-fold concentrated liposomes = 10× (concentrated using liposome extruder purification, LEP) (28). We previously observed that the 1× rapamycin dose increased mean lifespan more robustly (+11.4%) in the presence of an antibiotic (carbenicillin) (15); the antibiotic test was performed to exclude the possibility that rapamycin extends lifespan by reducing life-shortening infection by the *E. coli* food source. Seemingly, such infection can mask the effect of rapamycin on lifespan. Here again, drug effects on lifespan were tested with and without carbenicillin present.

**Figure 1.**
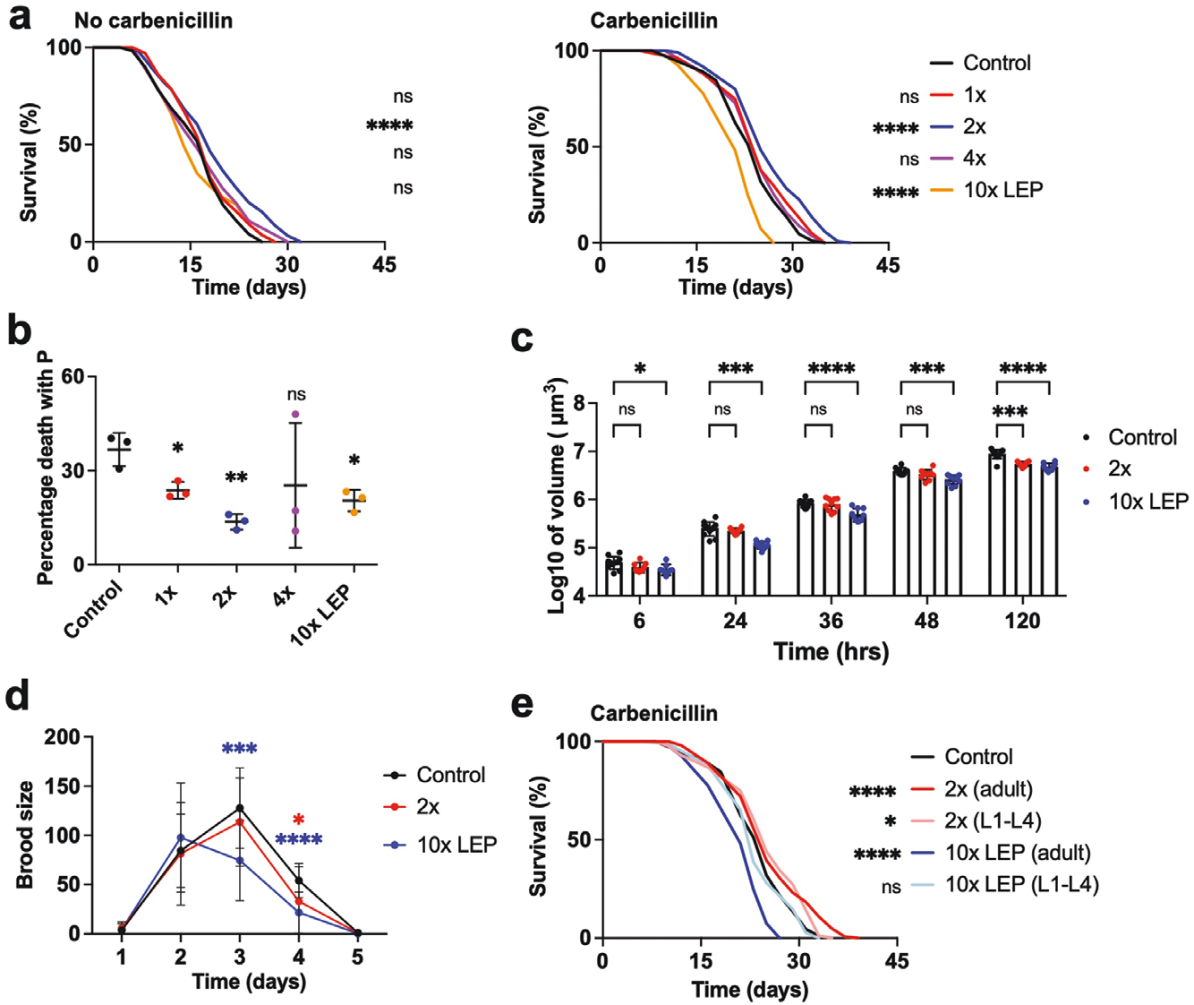
Effects of rapamycin on *C. elegans* life history. (**A**) Dose–dependent effects of rapamycin administered during adulthood on lifespan. Left, carbenicillin absent. Right, carbenicillin present. LEP, liposome extruder purification. Controls: empty liposomes (no drug). (**B**) Rapamycin reduces P death frequency (mortality with severe pharyngeal infection). (**C**) Rapamycin treatment during development retards growth. (**D**) Rapamycin treatment during development reduces progeny number. (**E**) Development-limited exposure to rapamycin (2× dose) can modestly increase lifespan (carbenicillin present). *p < .05, **p < .01, ***p < .001, ****p < .0001. (A), (E), log rank test; (C), (D) Tukey test. (E) Comparisons are to control (Supplementary Table 2).

Dose-dependent effects on lifespan were seen under both conditions (Figure 1A, Supplementary Figure 1A,B, Supplementary Tables 1 and 2). In the absence of carbenicillin, only 2× rapamycin significantly increased mean lifespan (+ 16.1%, *p *< .0001, log rank test; summed data from 3 trials, here and elsewhere unless otherwise stated). In the presence of carbenicillin, 1× rapamycin significantly increased lifespan in 2/3 trials (summed data: *p *= .069, log rank), 2× rapamycin significantly increased lifespan (+ 10.4, *p *< .0001, log rank) (Supplementary Table 2). Notably, the magnitude of the increases resulting from either dose were not greater than in the absence of carbenicillin, in contrast to our previous observations (15), for reasons that are unclear. In fact, the largest effect seen was in a trial with the 2× dose without carbenicillin (+21.9%, *p* = .0021, log rank) (Supplementary Table 1). By contrast, 10× rapamycin shortened lifespan (−12.1%, *p *< .0001, log rank, Figure 1A) in the presence but not the absence of carbenicillin, suggesting a protective effect of proliferative *E. coli* against rapamycin toxicity. We conclude that among these rapamycin dosages, the 2× dose is optimal for increasing lifespan.

### Rapamycin Reduces P Death Frequency and Increases p Lifespan

To better understand rapamycin effects on *C. elegans* cultured on live, proliferating bacteria (ie, without carbenicillin), we performed mortality deconvolution, which combines survival analysis with necropsy of worms after death from old age. In aging wild-type *C. elegans* populations, around 40% die with a swollen pharynx heavily infected with *E. coli*, so-called P (“big P”) deaths (29). For the remaining deaths, corpses have an atrophied pharynx, so-called p (“small p”) deaths. P deaths largely occur before p deaths, such that reducing the proportion of P deaths can increase the mean lifespan of the population. Antibiotic treatment extends overall lifespan (32), preventing P death and extending p lifespan (29).

Rapamycin significantly reduced the proportion of P deaths at 1×, 2×, and 10× doses (Figure 1B) but, as a rule, did not increase P lifespan (Supplementary Table 1). In principle, this could reflect an increase in infection resistance or an effect of rapamycin on bacterial pathogenicity (or both); rapamycin was previously shown not to reduce *E. coli* proliferation (2), but might still affect pathogenicity. A small but significant increase in p lifespan was seen with 2× rapamycin only (Supplementary Figure 1C; Supplementary Table 1). Thus, the increase in overall lifespan caused by 2× rapamycin results from a combination of a decrease in the proportion of P deaths and an increase in p lifespan. That p but not P lifespan is increased is evident in the increase in maximum but not median lifespan, resulting in a neck-and-shoulder type bimodal survival curve (29).

The lack of life-extension with the 4× and 10× treatments could reflect either rapamycin toxicity, or toxicity of the DMSO solvent which was present in liposomes at 0.5%. To assess whether DMSO might suppress life-extending effects of rapamycin at higher doses, nematodes were exposed to liposomes prepared as in the 10× rapamycin treatment, but containing either only 0.5% DMSO or water. DMSO did not significantly affect lifespan (−5.0%, *p* = .44, log rank; carbenicillin present) (Supplementary Figure 1D). This implies that it is the high-dose rapamycin itself that shortens lifespan. This could reflect either deleterious effects of severe mTOR inhibition or off-target (ie, mTOR-independent) effects of rapamycin on *C. elegans*.

### Rapamycin Treatment During Development Can Modestly Increase Lifespan

It is of interest to know how rapamycin administered at different ages affects lifespan, for several reasons: first, to discover at what stages mTOR signaling affects lifespan, and second to inform possible use of rapamycin as an antiaging therapy in mammals (possibly including humans). It was recently shown that rapamycin administration during development can increase lifespan both in *Drosophila* and mice (19–21). We set out to test whether a similar effect is seen in *C. elegans*.

We first tested effects on *C. elegans* growth of exposure to 2× or 10× rapamycin during development (from L1 to L4 stage). This resulted in inhibition of growth, evident as reduced worm volume at 120 hours of age, of 38.6% and 46.5%, respectively (Figure 1C; Supplementary Figure 2A). Inhibition of growth by rapamycin is consistent with lowered mTOR signaling, genetic inhibition of which leads to arrested development (33).

We also tested effects on fertility, which is reduced in fruit flies exposed to rapamycin during adulthood (34). In *C. elegans*, rapamycin treatment during development (L1–L4) reduced brood size, the 2× dose by 13.4% and the 10× dose by 26.7%, and the 10× dose caused an earlier peak in daily fecundity (Figure 1D, Supplementary Figure 2B); exposing adults to rapamycin was previously observed not to reduce brood size (2). After the end of egg laying due to self-sperm depletion, hermaphrodites lay large numbers of unfertilized oocytes (35), and this is strongly suppressed by mutation of the *daf-2* insulin/IGF-1 receptor (36). Similarly, 2× and 10× rapamycin treatment restricted to development caused a modest but statistically significant reduction in number of unfertilized oocytes laid, from 57.7 per worm to 50.2 and 42.0 per worm, that is, by 12.9% and 27.1%, respectively (Supplementary Figure 2C). Taken together, these results suggest that rapamycin exposure during development reduces oocyte production by adults.

Effects of 2× and 10× rapamycin exposure during the L1–L4 stages on lifespan was then tested. In three trials, 2× rapamycin caused modest, apparent increases in mean lifespan (+2.0%-7.2%) which did not reach statistical significance. However, summed data from the 3 trials showed a significant 4.6% increase in lifespan (*p* = .027, log rank). The 10× rapamycin treatment did not increase lifespan (Figure 1E, Supplementary Table 2), suggesting a possible detrimental effect of high-dose rapamycin on development.

### Late-Life Treatment With Rapamycin Robustly Increases Lifespan

Notably, mouse lifespan can be extended by rapamycin either from late middle age onwards (4), or briefly in later life (22,23). To be able to test this in *C. elegans*, we first established the efficacy of liposome-mediated compound delivery at later ages. One concern here is that feeding (pharyngeal pumping) rate declines with age in *C. elegans*, particularly after Day 7 of adulthood (37), which is expected to reduce drug ingestion.

**Figure 2.**
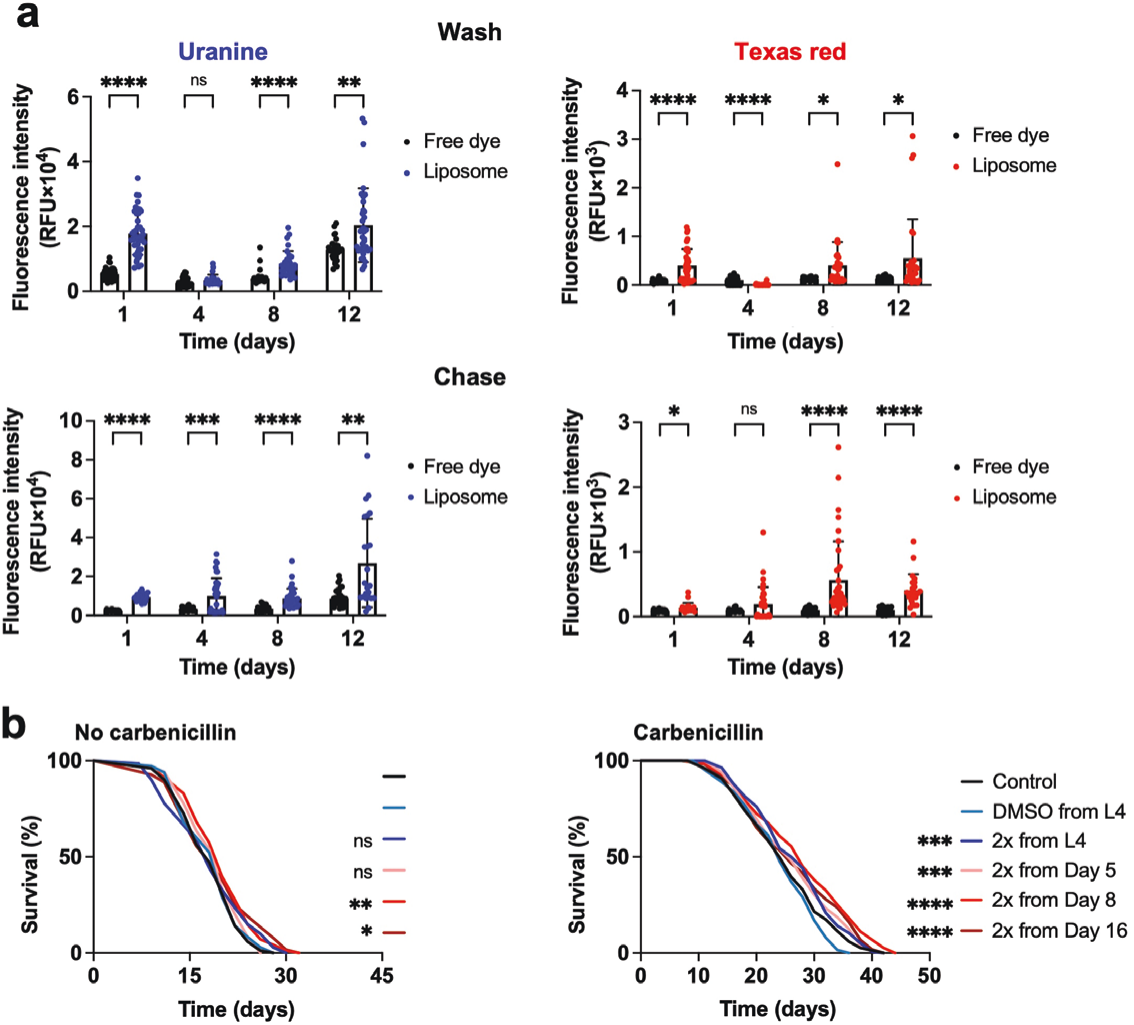
Effects of administration of 2× rapamycin at later ages. (**A**) Liposome-mediated compound delivery remains efficient up to Day 12 of adulthood. Top, wash treatment. Below, chase treatment. (**B**) Rapamycin increases lifespan more when administered starting at later ages. Left, no carbenicillin; right, carbenicillin added. *p < .05, **p < .01, ***p < .001, ****p < .0001. (A), Šidák test, (B), log rank test, compared to DMSO control.

To test this concern, we exposed older animals for 3 hours to liposomes containing 2 fluorescent dyes: uranine (green fluorescence, good membrane permeability) and Texas red (red fluorescence, poor membrane permeability). Fluorescence in nematodes was then examined with or without a chase (incubation on a dye-free bacterial lawn to flush dye from the intestinal lumen). This assayed dye uptake into nematode tissue excluding or including, respectively, dye present in the alimentary canal (15). Nematodes were exposed to dye-loaded liposomes on Days 1, 4, 8 and 12 of adulthood. Both with and without chase treatments, uptake of uranine remained high until Day 12, and use of liposomes significantly increased uranine uptake (Figure 2A). Thus, liposome-mediated compound delivery remains effective until at least day 12 of adulthood, that is, up to ~60% of mean lifespan (equivalent to ~50 years in humans, given a mean *C. elegans* lifespan of 18 days, and a typical developed world human life expectancy of ~82 years).

Next we tested effects on lifespan of 2× rapamycin administration commencing at different ages (L4, Days 5, 8, and 16 of adulthood) with or without carbenicillin present. Our initial expectation was that the magnitude of effects of rapamycin on lifespan would decrease with administration starting from progressively later ages. However, this proved not to be the case. In the absence of carbenicillin, 2× rapamycin increased lifespan only when administered from Days 8 and 16 of adulthood, by +8.5%, *p* = .0039 and + 4.5%, *p* = .018 (log rank), respectively (Figure 2B, left; Supplementary Table 3). This suggests greater effects given delivery at later ages, though the lack of an effect with administration from L4 onwards is at odds with earlier trials (Figure 1A, left). In the presence of carbenicillin, rapamycin increased lifespan in all cases, with the largest effect seen given administration from Day 8 onwards (+15.1%, *p* < .001, log rank), and only in this case was a statistically significant increase in lifespan seen in all 3 trials (Figure 2B, right; Supplementary Table 4).

In the case of treatment from Day 16, some animals had died of old age prior to rapamycin administration. To assess survival after this time point, earlier deaths were censored. Mean lifespan from Day 16 was increased by rapamycin administration by +17.0% *p* < .0001, log rank (Supplementary Table 4).

Taken together, these results show, surprisingly, that rapamycin administered to *C. elegans* later in life if anything causes larger rather than smaller increases in lifespan. These life-extending effects suggest the occurrence of life-shortening processes operative in later life that rapamycin ameliorates.

### Temsirolimus Increases *C. elegans* Lifespan

A goal of this study was to establish a protocol for robust and reproducible extension of *C. elegans* lifespan by pharmacological inhibition of mTOR. Although we have achieved this, the magnitude of effects on mean lifespan (maximally +21.9%) is still relatively modest. One possibility is that rapamycin’s low solubility limits bioavailability. To explore this we assessed effects on *C. elegans* of two widely used derivatives of rapamycin (rapalogs) that have greater solubility, everolimus and temsirolimus (18).

**Figure 3.**
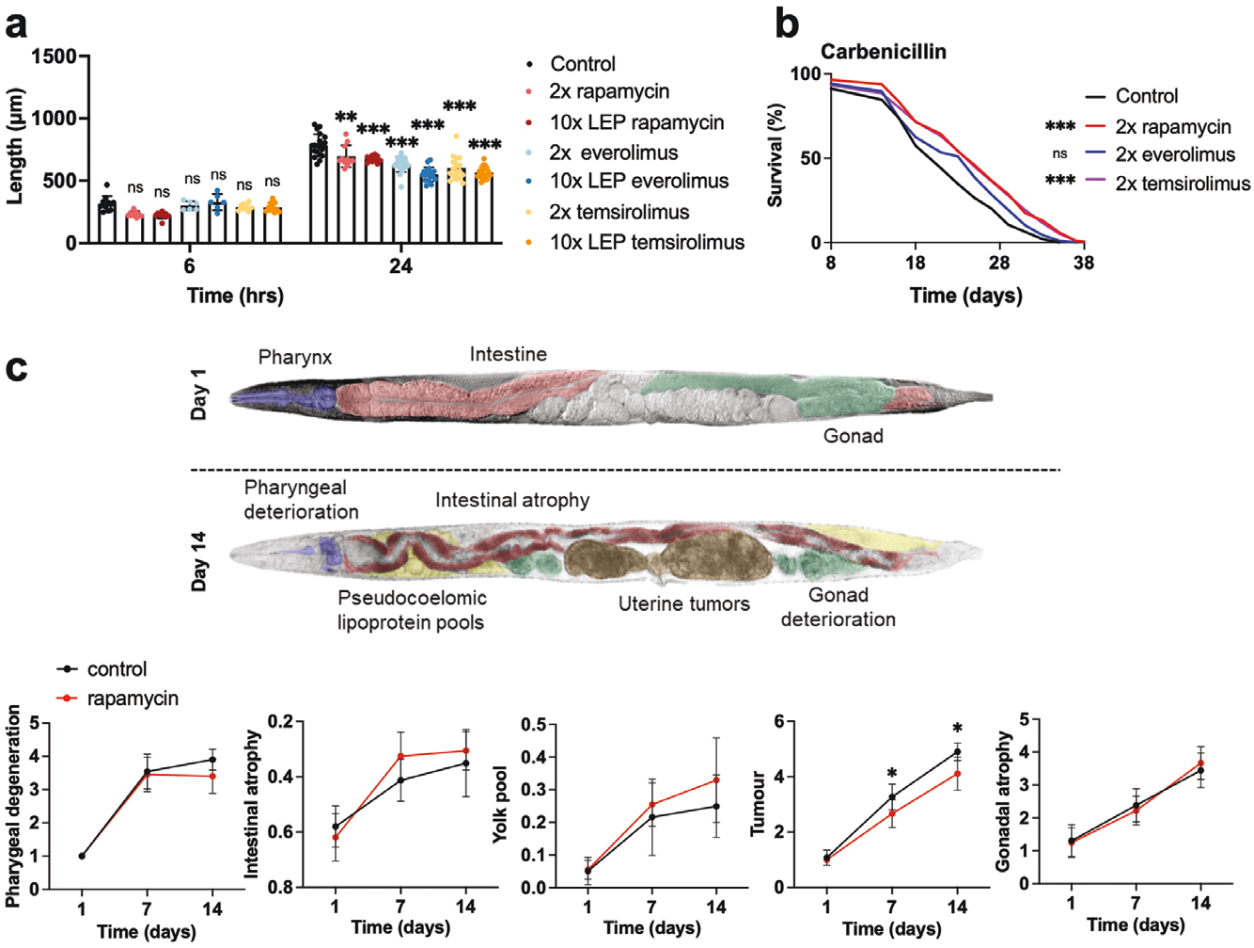
(**A,B**) Effects of rapalog administration on *C. elegans*. (A) Rapalogs retard *C. elegans* growth. The specified measurement times are approximations. (B) Temsirolimus extends *C. elegans* lifespan; carbenicillin present. (**C**) Effects of rapamycin on age-related pathology (segmental antigeroid effect); no carbenicillin. *p < .05, **p < .01, ***p < .001, ****p < .0001. (A), (C) Šidák test; (B) log rank test.

As a test of drug uptake, effects of rapalogs on development were assayed at the 2× and 10× doses. Both rapalogs reduced growth at the 2 concentrations, evident after 24 hours (Figure 3A). At the 2× dose rapamycin and the 2 rapalogs inhibited growth to a similar extent. This suggests that rapamycin and its derivatives behave similarly in *C. elegans* in terms of drug uptake and inhibition of mTOR.

Next, we compared the capacity of rapamycin and the 2 rapalogs to extend lifespan, using the 2× dose that is optimal for rapamycin, with carbenicillin present. Drug administration was started on Day 8 of adulthood, given the relatively strong effects of rapamycin administered from this age onwards (Figure 2B). Rapamycin again significantly increased lifespan in 3/3 trials (+15.0%, *p* = .0002, log rank, summed data) (Figure 3B, Supplementary Table 5). Temsirolimus caused increases in lifespan that reached statistical significance in 1/3 trials, but the summed data showed a significant effect similar in magnitude to that of rapamycin (+13.9%, *p *= .0005, log rank). By contrast, despite a consistent trend toward increased lifespan, everolimus had no significant effect (+6.8%, *p* = .075, log rank, summed data) (Figure 3B, Supplementary Table 5).

Taken together, these results suggest that the 2 rapalogs are taken up by *C. elegans* and inhibit mTOR, and that effects of temsirolimus on lifespan are similar to those of rapamycin, but those of everolimus are weaker.

### Mixed Effects of Rapamycin on Age-Related Pathologies

In mice, rapamycin ameliorates many but not all diseases of aging and in a few cases worsens them (24,25). To test whether the same is true of *C. elegans*, we examined effects on several prominent, naturally occurring and well-characterized age-related pathologies that develop in mid-life in *C. elegans* hermaphrodites. These are deterioration of the pharynx, atrophy of the intestine, pseudocoelomic lipoprotein pool (PLP) accumulation, atrophy and fragmentation of the distal gonad, and development of teratoma-like uterine tumors (30–32,38) (Figure 3c).

Rapamycin was administered at the 2× dose from L4 onwards and effects on Days 1, 7, and 14 of adulthood assayed (no carbenicillin). Drug treatment significantly ameliorated one pathology: uterine tumor size was reduced (Figure 3C). No effect was seen on pharyngeal deterioration or distal gonad atrophy, although intestinal atrophy and PLP accumulation appeared to be worsened at the 2 later time points, though effects did not reach statistical significance (Figure 3C); the apparent effect on the intestine is reminiscent of *let-363* (mTOR) mutants, in which arrested larvae exhibit intestinal atrophy (33). Thus, as in mice, the effects of rapamycin on age-related pathology in *C. elegans* are mixed.

## Discussion

This study builds upon earlier studies of rapamycin on *C. elegans* (2,15,39) to provide a fuller picture of the effects of this important antiaging drug. New findings include identification of a rapamycin dose with maximal effects on nematode lifespan, use of mortality deconvolution data to elucidate anti-infection effects of rapamycin, life extension resulting from treatment restricted to development or, particularly, to later life, life extension by temsirolimus but not everolimus, and mixed effects of rapamycin on age-related pathologies.

### Effects of Rapamycin Treatment at Different Doses

We had hoped that delivery of rapamycin at higher doses or use of rapalogs would lead to larger increases in lifespan, that would increase analytical power in future studies of rapamycin and *C. elegans* aging. The largest increase in mean lifespan that we were able to achieve was 21.9%, using the 2× rapamycin dose (Figure 1A).

One implication of our findings is that life-extending effects of rapamycin are limited by drug toxicity at higher doses (Figure 1A). If correct, this might be an idiosyncrasy of *C. elegans*. In mammals rapamycin toxicity is very low. The LD50 for rapamycin in rats is so high (> 2 500 mg/kg) that it is not possible to determine (40). Low toxicity in humans is suggested by the consequences of a suicide attempt in which an individual consumed 103 1 mg rapamycin tablets, with no apparent harmful effects (41). Short-term use of rapamycin in humans does have some side-effects, in the main relatively mild (40), but long-term administration in mice can have more serious effects (testicular atrophy and increased cataract frequency) (24). The life-shortening effects of high dose rapamycin in *C. elegans* could reflect pathologically low levels of mTOR signaling, or unrelated, toxic off-target effects. The doses of rapamycin that increase lifespan in *C. elegans* are already far higher than those used in mammals (2).

The effects of rapamycin on lifespan varied somewhat between trials, for unknown reasons. In *Drosophila*, the extent of life extension by rapamycin can vary greatly between strains (34); however, in the present study, the same isogenic N2 strain was used in all trials. A previous multi-lab comparison of drug effects on *C. elegans* lifespan described marked variability within labs over time, rather than between labs (42). Our results are consistent with this: for most experiments, where three trials were performed in quick succession, there was relatively little variability. By contrast, the differences seen were between experiments performed with large time intervals between them. A further issue is that it could be argued that the variability of drug effects means that a more stringent cutoff to define statistical significance should be applied; moreover, we note that although for each lifespan test 3 trials were conducted, the sample sizes used were relatively small.

### Effects of Rapamycin Treatment at Different Ages

As in fruit flies and mice (19–21), rapamycin exposure limited to development or later life was sufficient to extend nematode lifespan (Figures 1E and 2B). These findings are consistent with the proposed hypothesis (43) that there exist 2 distinct windows for rapamycin action in aging, one during development and one during adulthood. However, given the very short lifespan of *C. elegans*, it is possible that after exposure limited to the developmental period, rapamycin perdures within the adult worm, and continues to inhibit mTOR.

The effects on lifespan of rapamycin treatment restricted to later life were, if anything, greater than those of rapamycin throughout life (Figure 2B, Supplementary Figure 3). On the face of it, this is paradoxical given that under both conditions rapamycin is present in later life. One possibility is this relates to the rapamycin pharmacokinetics. Aging causes substantial changes in drug metabolism in humans (44). In mammals rapamycin is metabolized by the action of cytochrome P450 oxidases of the *CYP3A* family (45). Possibly exposure of *C. elegans* to rapamycin induces expression of CYPs that metabolize the drug, and this induction occurs more readily in younger animals, such that if rapamycin treatment is initiated only in later life, the drug is less quickly metabolized.

Rapamycin treatment initiated in late life robustly increased lifespan, even from Day 16 (Figure 2B, Supplementary Figure 3), which is long after maximal development of the major age-related pathologies listed above. This implies that life-extending effects of rapamycin are, at least to some extent, exerted via age changes occurring after the mid-life pathogenetic burst (30). Moreover, of these pathologies only uterine tumor development was suppressed by rapamycin (Figure 3C). Possibly suppression of tumor development contributes to effects on lifespan; however, under otherwise standard culture conditions, full suppression of tumor growth using the anticancer drug 5-fluorodeoxyuridine (floxuridine) does not increase *C. elegans* lifespan (46).

### Rapamycin has Segmental Antigeroid Effects

The mixed effect of rapamycin on age-related pathology seen here recapitulate findings in the mouse (24,25). The implications of these findings are interesting, as follows. The occurrence of changes in rate of appearance in some but not all age-related pathologies is a feature of human genetic diseases that resemble accelerated aging. The renowned gerontologist George Martin (1927–2022) described such conditions as either *segmental progeroid* syndromes, where several but not all features of aging are accelerated (eg, Werner syndrome), or *unimodal progeroid* syndromes where only a single feature is accelerated (eg, early onset familial Alzheimer’s disease) (47).

More recently, in 2016, Martin and colleagues introduced the concept of *unimodal antigeroid* conditions, where a single aspect of aging is decelerated (48). Here a possible human example is isolated growth hormone (GH) deficiency due to loss of the GH releasing hormone receptor, which causes apparent cancer resistance but has little effect on lifespan (49). A natural completion of this typology, that we propose here, is the term *segmental antigeroid* to describe cases where several but not all features of aging are decelerated. This term is applicable to effects of rapamycin. However, to be precise, the effect of rapamycin in mice, and perhaps *C. elegans* too, is simultaneously segmental antigeroid and segmental progeroid, with the former effect predominating. Notably, rapamycin reduces frequency of death with pharyngeal infection, and uterine tumor size, although not improving several other age-related pathologies (Figures 1B and 3C).

## Supplementary Material

glae196_suppl_Supplementary_Materials

glae196_suppl_Supplementary_Tables
