# Supplementary material for: Characterization of Effects of mTOR Inhibitors on Aging in Caenorhabditis elegans
Source: J Gerontol A Biol Sci Med Sci. 2024 Aug 16;79(11):glae196. doi: 10.1093/gerona/glae196 (PMC11374883; doi:10.1093/gerona/glae196)
Supplement: glae196_suppl_Supplementary_Materials [file glae196_suppl_supplementary_materials.pdf]

Supplementary material

Supplementary Figure 1. Details of effects of rapamycin on *C. elegans* lifespan.

Supplementary Figure 2. Details of effects of rapamycin on development and fertility.

Supplementary Figure 3. Effects of rapamycin on lifespan when administered at later ages, individual trials.

Supplementary Table 1. Tests of effects of different doses of rapamycin on *C. elegans* lifespan (no carbenicillin).

Supplementary Table 2. Tests of effects of different doses of rapamycin on *C. elegans* lifespan (carbenicillin present).

Supplementary Table 3. Effects of rapamycin treatment commencing at different ages (no carbenicillin).

Supplementary Table 4. Effects of rapamycin treatment commencing at different ages (carbenicillin present).

Supplementary Table 5. Effects of rapamycin and rapalogs on lifespan.

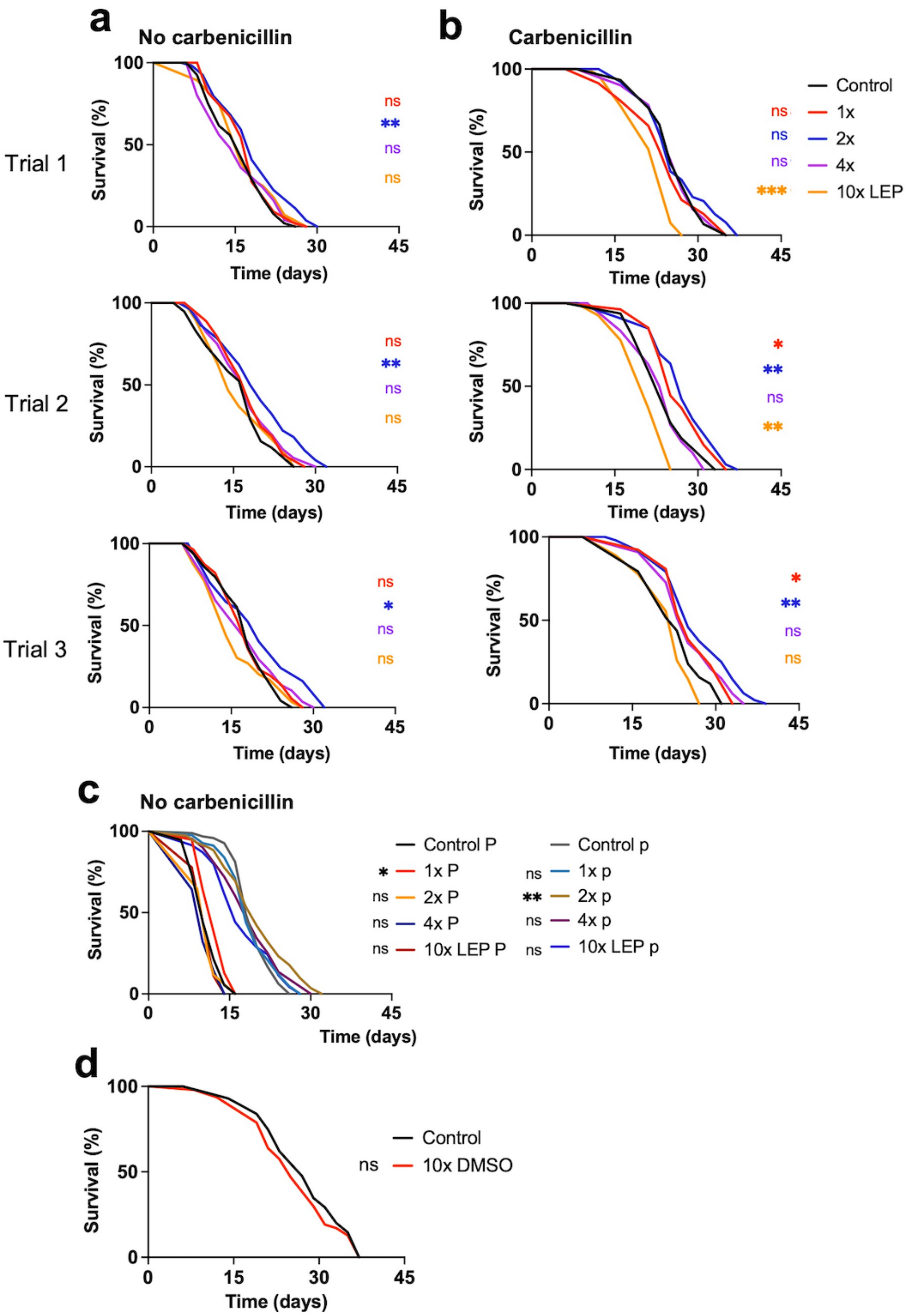

Supplementary Figure 1. Details of effects of rapamycin on *C. elegans* lifespan. Individual trials testing dose-dependent effects on lifespan of rapamycin administered during adulthood. (a) Carbenicillin absent. (b) Carbenicillin present. (c) Effects of rapamycin on P and p lifespan (two forms of death; mortality deconvolution). For details of statistics, see Supplementary Table 1. (d) No effect on lifespan of liposomes containing 5% DMSO only, equivalent in quantity to 10x rapamycin dose ($N = 1$; control: $n = 55$ (10 censored); DMSO treatment: $n = 47$ (1 censored). $^{*}p < 0.05$, $^{**}p < 0.01$, $^{***}p < 0.001$, log rank test.

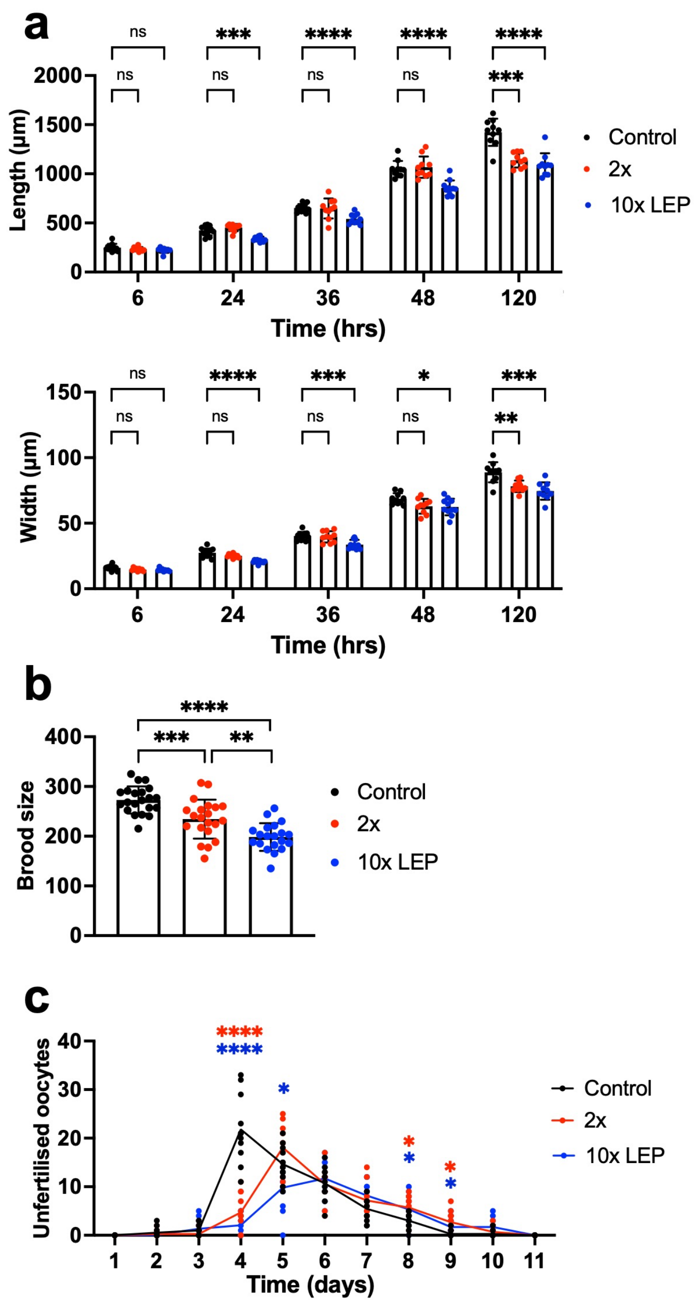

Supplementary Figure 2. Details of effects of rapamycin treatment during development (L1-L4) on development and fertility. (a) Reduction in nematode size. Top, length. Bottom, width. (b) Reduction in offspring number. (c) Delayed production and decreased quantity of unfertilized oocytes laid (n = 11 broods). *p < 0.05, **p < 0.01, ***p < 0.001, ****p < 0.0001, Tukey test.

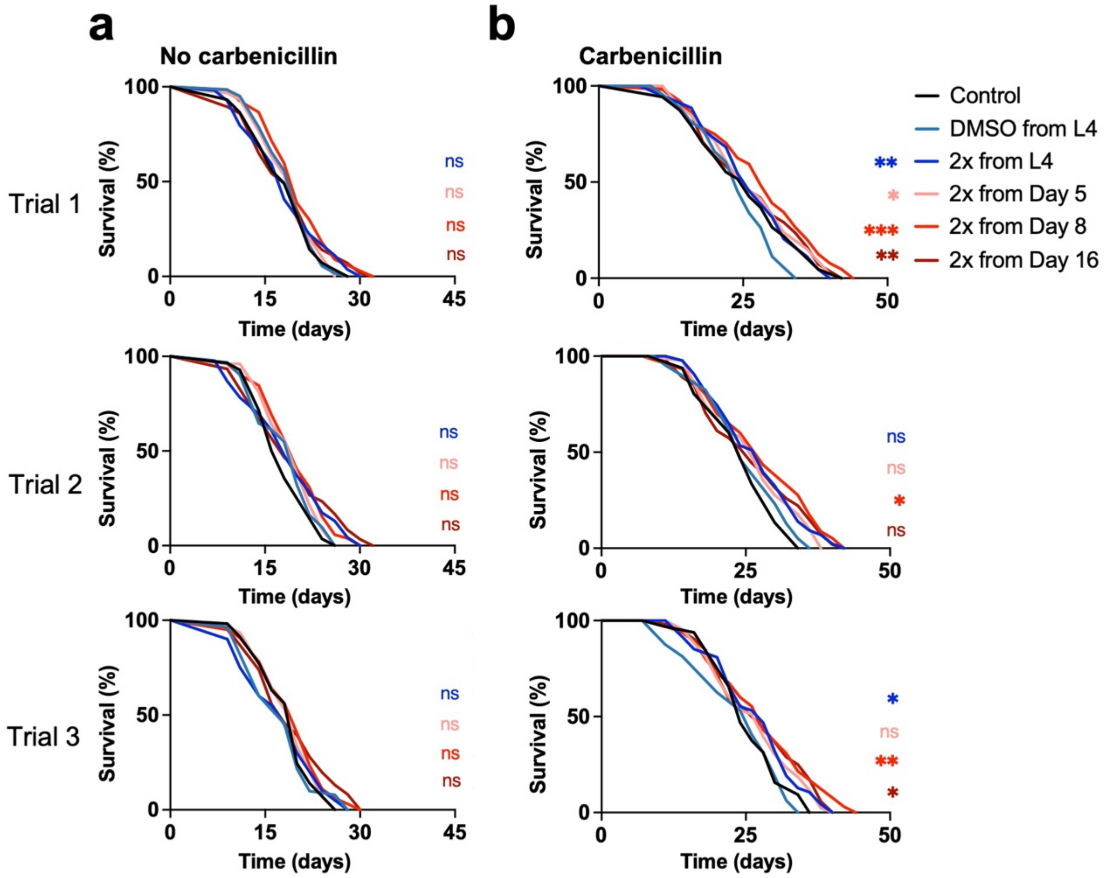

Supplementary Figure 3. Effects of rapamycin on lifespan when administered at later ages, individual trials. (a) No carbenicillin. (b) Carbenicillin added. For details of statistics, see Supplementary Tables 3 and 4. *$p < 0.05$, **$p < 0.01$, ***$p < 0.001$, log rank test compared to DMSO + carbenicillin.

Supplementary Table 1. Tests of effects of different doses of rapamycin on *C. elegans* lifespan (no carbenicillin)

Strain/ condition	Number of deaths/ censored	All death			P death				p death		
		Mean lifespan (days)	% change vs. control	*p* vs. control (log rank)	%P of death	Mean lifespan (days)	% change vs. control	*p* vs. control (log rank)	Mean lifespan (days)	% change vs. control	*p* vs. control (log rank)
Control (50 μL liposomes, no drug)	**[C] 153/32**	**16.27**			**37.25**	**10.79**			**19.33**		
	[1] 52/9	15.67			40.38	10.90			18.45		
	[2] 52/8	15.80			40.38	10.10			19.55		
	[3] 49/15	17.40			30.61	11.60			19.94		
1x rapamycin (50 μL liposomes, 100 μM)	**[C] 164/30**	**17.34**	**+6.58**	**0.0743**	**23.78**	**12.21**	**+13.16**	**0.0174**	**18.94**	**-2.02**	**0.7489**
	[1] 55/13	16.80	+7.21	0.3127	21.82	12.00	+10.09	0.2047	18.14	-1.68	0.9859
	[2] 53/9	17.53	+10.95	0.1735	22.64	13.00	+28.71	0.0073	18.83	-3.68	0.9556
	[3] 56/8	17.71	+1.78	0.4750	26.79	11.73	+1.12	0.9304	19.90	-0.20	0.5559
2x rapamycin (100 μL liposomes, 100 μM)	**[C] 129/19**	**18.89**	**+16.10**	**<0.0001**	**13.18**	**10.88**	**+0.83**	**0.5672**	**20.06**	**+3.78**	**0.0093**
	[1] 54/7	18.29	+16.72	0.0085	11.11	10.33	-5.23	0.2206	19.27	+4.44	0.1726
	[2] 50/8	19.27	+21.96	0.0021	14.00	11.57	+14.55	0.2813	20.47	+4.71	0.0772
	[3] 25/4	19.41	+11.55	0.0412	16.00	10.50	-9.48	0.2370	21.05	+5.57	0.0783
4x rapamycin (200 μL liposomes, 100 μM)	**[C] 139/11**	**16.95**	**+4.18**	**0.0627**	**20.14**	**10.21**	**-5.38**	**0.2261**	**18.65**	**-3.52**	**0.5343**
	[1] 25/3	15.44	-1.47	0.6477	48.00	10.00	-8.26	0.2799	20.46	+10.89	0.0972
	[2] 56/5	17.39	+10.06	0.1661	10.71	9.67	-4.26	0.7104	18.32	-6.29	0.6650
	[3] 58/3	17.17	-1.32	0.4705	17.24	10.80	-6.90	0.3451	18.50	-7.22	0.8251
10x rapamycin (100 μL 5x concentrated liposomes, 100 μM)	**[C] 88/6**	**16.02**	**-1.54**	**0.7001**	**20.45**	**10.67**	**-1.11**	**0.5951**	**17.37**	**-10.14**	**0.3310**
	[1] 28/0	16.57	+5.74	0.3056	21.43	11.00	+0.92	0.9762	18.09	-1.95	0.6747
	[2] 30/2	16.00	+1.26	0.7433	16.67	10.40	+2.97	0.8617	17.12	-12.43	0.3045
	[3] 30/4	15.54	-10.69	0.4363	23.33	10.57	-8.88	0.3123	16.96	-14.94	0.2723

Drug from L4 onwards, 20˚C. Columns 2-5: lifespan of entire populations. Columns 6-12: data after mortality deconvolution. Columns 6-9: data for P sub-populations. Columns 10-12: data for p sub-populations.

Supplementary Table 2. Tests of effects of different doses of rapamycin on *C. elegans* lifespan (carbenicillin present)

**Strain/ condition** (all treatments with carbenicillin added)	**Number of deaths/ censored**	**Mean lifespan (days)**	**% change vs. control**	***p* vs. control (log rank)**
Control (50 μL liposomes, no drug)	**[C] 89/6**<br>[1] 30/2<br>[2] 32/1<br>[3] 27/3	**24.05**<br>25.53<br>23.69<br>22.91		
1x rapamycin (50 μL liposomes, 100 μM)	**[C] 100/15**<br>[1] 47/4<br>[2] 27/4<br>[3] 26/7	**25.00**<br>23.87<br>26.44<br>25.54	**+3.95**<br>-6.50<br>-11.61<br>+11.48	**0.0693**<br>0.6132<br>0.0368<br>0.0475
2x rapamycin (100 μL liposomes, 100 μM)	**[C] 120/21**<br>[1] 39/9<br>[2] 33/5<br>[3] 48/7	**26.57**<br>26.10<br>27.09<br>26.58	**+10.48**<br>+2.23<br>+14.35<br>+16.02	**<0.0001**<br>0.3744<br>0.0046<br>0.0031
4x rapamycin (200 μL liposomes, 100 μM)	**[C] 114/17**<br>[1] 51/7<br>[2] 30/6<br>[3] 33/4	**24.82**<br>25.33<br>23.50<br>25.21	**+3.20**<br>-0.78<br>-0.80<br>+10.04	**0.2287**<br>0.9833<br>0.7760<br>0.0691
10x rapamycin (100 μL 5x concentrated liposomes, 100 μM)	**[C] 81/11**<br>[1] 27/5<br>[2] 27/3<br>[3] 27/3	**21.15**<br>21.52<br>20.70<br>21.22	**-12.06**<br>-15.71<br>-12.62<br>-7.38	**<0.0001**<br>0.0002<br>0.0068<br>0.1752
2x rapamycin from L1-L4 (100 μL liposomes, 100 μM)	**[C] 84/11**<br>[1] 23/8<br>[2] 31/2<br>[3] 30/1	**25.16**<br>26.04<br>25.06<br>24.57	**+4.62**<br>+2.00<br>+5.78<br>+7.25	**0.0270**<br>0.6139<br>0.1187<br>0.0741
10x rapamycin from L1-L4 (100 μL 5x concentrated liposomes, 100 μM)	**[C] 75/6**<br>[1] 29/2<br>[2] 25/3<br>[3] 21/1	**23.74**<br>23.55<br>25.08<br>22.42	**-1.29**<br>-7.76<br>+5.87<br>-2.14	**0.6488**<br>0.1001<br>0.2737<br>0.6573

20˚C. Rows 2-6: treatment from L4. Rows 7-8: treatment during development only.

Supplementary Table 3. Effects of rapamycin treatment commencing at different ages (no carbenicillin)

Strain/ condition	Number of deaths/ censored	All deaths					Number of deaths/ censored	Deaths after day 16				
		Mean lifespan (days)	% change vs. control	*p* vs. control (log rank)	% change vs. DMSO	*p* vs. DMSO (log rank)		Mean lifespan (days)	% change vs. control	*p* vs. control (log rank)	% change vs. DMSO	*p* vs. DMSO (log rank)
Control (liposomes, no drug)	**[C] 171/1**<br>[1] 58/1<br>[2] 56/0<br>[3] 57/0	**18.19**<br>18.09<br>17.93<br>18.54										
Control (liposomes, 0.5% DMSO only)	**[C] 175/4**<br>[1] 60/2<br>[2] 62/0<br>[3] 53/2	**18.24**<br>18.95<br>18.42<br>17.97	**+0.27**<br>+4.75<br>+2.73<br>-3.07	**0.5440**<br>0.6657<br>0.4848<br>0.8869								
2x rapamycin from L4 stage	**[C] 140/0**<br>[1] 54/0<br>[2] 46/0<br>[3] 40/0	**18.21**<br>18.28<br>18.61<br>17.55	**+0.11**<br>+1.05<br>+3.79<br>-5.34	**0.1308**<br>0.4603<br>0.1053<br>0.9831	**-0.16**<br>-3.54<br>+1.03<br>-2.34	**0.3537**<br>0.7340<br>0.2581<br>0.8462						
2x rapamycin from day 5	**[C] 167/6**<br>[1] 60/0<br>[2] 47/4<br>[3] 60/2	**18.91**<br>18.80<br>19.18<br>18.81	**+3.96**<br>+3.92<br>+6.97<br>+1.46	**0.1947**<br>0.6922<br>0.1965<br>0.6953	**+3.96**<br>-0.79<br>+4.13<br>+4.67	**0.5600**<br>0.9485<br>0.5878<br>0.6232						
2x rapamycin from day 8	**[C] 183/0**<br>[1] 67/0<br>[2] 52/0<br>[3] 64/0	**19.73**<br>20.19<br>19.75<br>19.28	**+8.47**<br>+11.61<br>+10.15<br>+3.99	**0.0006**<br>0.0257<br>0.0218<br>0.1666	**+8.47**<br>+6.54<br>+7.22<br>+7.29	**0.0039**<br>0.0588<br>0.1251<br>0.1244						
2x rapamycin from day 16	**[C] 178/0**<br>[1] 31/0<br>[2] 60/0<br>[3] 61/0	**19.06**<br>18.53<br>19.43<br>19.18	**+4.78**<br>+2.43<br>+8.37<br>+3.45	**0.0057**<br>0.3305<br>0.0348<br>0.1304	**+4.49**<br>-2.22<br>+5.48<br>+6.73	**0.0184**<br>0.5897<br>0.0675<br>0.1104	**[C] 99/0**<br>[1] 31/0<br>[2] 34/0<br>[3[34/0	**23.49**<br>23.10<br>23.82<br>23.52	**+9.15**<br>+5.96<br>+10.43<br>+10.84	**<.0001**<br>0.0920<br>0.0083<br>0.0026	**+8.90**<br>+7.84<br>+9.87<br>+8.89	**<.0001**<br>0.0272<br>0.0036<br>0.0392

2x treatment is 100 μl liposomes containing 100 μM rapamycin.

Supplementary Table 4. Effects of rapamycin treatment commencing at different ages (carbenicillin present)

Strain/ condition	Number of deaths/ censored	All deaths					Number of deaths/ censored	Deaths after addition of rapamycin				
		Mean lifespan (days)	% change vs. control	*p* vs. control (log rank)	% change vs. DMSO	*p* vs. DMSO (log rank)		Mean lifespan (days)	% change vs. control	*p* vs. control (log rank)	% change vs. DMSO	*p* vs. DMSO (log rank)
Control	**[C] 94/6**	**24.16**										
(liposomes,	[1] 32/2	25.34										
no drug)	[2] 30/4	23.98										
	[3] 32/0	23.13										
Control	**[C] 124/12**	**24.23**	**+0.28**	**0.8525**								
(liposomes,	[1] 32/2	23.63	-6.75	0.4162								
0.5% DMSO	[2] 39/8	25.15	+4.88	0.3146								
only)	[3] 53/2	23.92	+3.42	0.7807								
2x rapamycin	**[C] 134/14**	**26.81**	**+10.97**	**0.0011**	**+10.64**	**0.0003**						
from L4	[1] 47/4	26.85	+5.96	0.1553	+13.62	0.0428						
stage	[2] 43/5	26.84	+11.93	0.0343	+6.72	0.1453						
	[3] 44/5	26.73	+15.56	0.0316	+11.75	0.0092						
2x rapamycin	**[C] 130/13**	**26.37**	**+9.15**	**0.0026**	**+8.83**	**0.0008**	**[C]130/10**	**26.37**	**+9.15**	**0.0026**	**+8.83**	**0.0008**
from day 5	[1] 43/6	26.51	+4.62	0.2149	+12.18	0.0520	[1] 43/3	26.51	+4.62	0.2149	+12.19	0.0520
	[2] 41/4	26.10	+8.84	0.0618	+3.78	0.2212	[2] 41/4	26.10	+8.84	0.0618	+3.78	0.2212
	[3] 46/3	26.48	+14.48	0.0354	+10.70	0.0122	[3] 46/3	26.48	+14.48	0.0354	+10.70	0.0122
2x rapamycin	**[C] 188/9**	**27.88**	**+15.40**	**<.0001**	**+15.06**	**<.0001**	**[C] 188/2**	**27.88**	**+15.40**	**<.0001**	**+15.06**	**<.0001**
from day 8	[1] 65/5	27.72	+9.39	0.0436	+17.31	0.0070	[1] 65/0	27.72	+9.39	0.0436	+17.31	0.0070
	[2] 59/3	27.60	+15.10	0.0048	+9.74	0.0167	[2] 59/1	27.60	+15.10	0.0048	+9.74	0.0167
	[3] 64/1	28.30	+22.35	0.0012	+18.31	0.0001	[3]64/1	28.30	+22.35	0.0012	+18.31	0.0001
2x rapamycin	**[C] 161/7**	**26.52**	**+9.77**	**0.0005**	**+9.45**	**<.0001**	**[C] 140/0**	**28.36**	**+17.38**	**<.0001**	**+17.04**	**<.0001**
from day 16	[1] 52/3	27.22	+7.42	0.0640	+15.19	0.0120	[1] 47/0	28.60	+12.87	0.0141	+21.03	0.0021
	[2] 54/4	26.00	+8.42	0.0523	+3.38	0.1430	[2] 46/0	28.04	+16.93	0.0035	+11.49	0.0164
	[3] 55/0	26.28	+13.62	0.0237	+9.87	0.0079	[3]47/0	28.43	+22.91	0.0019	+18.85	0.0002

2x treatment (100 μl liposomes containing 100 μM rapamycin).

Supplementary Table 5. Effects rapamycin and rapalogs on lifespan (carbenicillin present)

Strain/ condition	Number of deaths/ censored	All death		
		Mean lifespan (days)	% change vs. control	*p* vs. control (log rank)
Control (liposomes, no drug)	**[C] 98/6**<br>[1] 24/3<br>[2] 25/1<br>[3] 49/2	**21.79**<br>21.65<br>23.00<br>21.26		
2x rapamycin from day 8	**[C] 100/13**<br>[1] 23/2<br>[2] 28/3<br>[3] 49/8	**25.05**<br>24.81<br>26.79<br>24.16	**+14.96**<br>+14.60<br>+16.48<br>+13.64	**0.0002**<br>0.0460<br>0.0385<br>0.0115
2x everolimus from day 8	**[C] 89/7**<br>[1] 27/2<br>[2] 31/2<br>[3] 31/3	**23.28**<br>22.93<br>23.77<br>23.12	**+6.84**<br>+5.91<br>+3.35<br>+8.75	**0.0750**<br>0.2587<br>0.6570<br>0.1366
2x temsirolimus from day 8	**[C] 95/8**<br>[1] 25/2<br>[2] 27/5<br>[3] 43/1	**24.81**<br>23.26<br>26.38<br>24.69	**+13.86**<br>+7.44<br>+14.70<br>+16.13	**0.0005**<br>0.1860<br>0.0508<br>0.0050

2x treatment (100 µl liposomes containing 100 µM drug) from day 8 of adulthood onwards, with carbenicillin present.
